# Supplementary material for: A Systematic Review of Prognostic Factors in Patients with Cancer Receiving Palliative Radiotherapy: Evidence-Based Recommendations
Source: Cancers (Basel). 2024 Apr 25;16(9):1654. doi: 10.3390/cancers16091654 (PMC11083084; doi:10.3390/cancers16091654)
Supplement: Supplementary file 1 [file cancers-16-01654-s001.zip › supplymentary materials/Table S1-Search methodology.docx]

**Table S1.** Search strategy.

| **#** | **Searches** | **Results** |
| --- | --- | --- |
| 1 | prognostication.ab,ti. | 10,921 |
| 2 | exp Prognosis/ | 1,876,583 |
| 3 | “prognos*”.ab,ti. | 747,185 |
| 4 | “prognostic factor*”.ab,ti. | 119,530 |
| 5 | 1 or 2 or 3 or 4 | 2,258,004 |
| 6 | exp Neoplasms/or (oncolog* or cancer* or carcinoma* or tumor* or tumour* or neoplasm* or metasta* or malignan*).mp. | 5,137,744 |
| 7 | exp Survival Analysis/ | 331,297 |
| 8 | exp Cancer Survivors/ | 7923 |
| 9 | “cancer survivor*”.ab,ti. | 22,353 |
| 10 | 7 or 8 or 9 | 354,421 |
| 11 | exp Clinical Decision-Making/ | 14,637 |
| 12 | “clinical decision making”.ab,ti. | 23,105 |
| 13 | 11 or 12 | 35,713 |
| 14 | 5 and 6 and 10 and 13 | 952 |
| 15 | exp Radiotherapy/ | 204,101 |
| 16 | radiotherapy.ab,ti. | 194,512 |
| 17 | “radiation therapy”.ab,ti. | 85,679 |
| 18 | “radiation treatment”.ab,ti. | 9995 |
| 19 | 15 or 16 or 17 or 18 | 366,767 |
| 20 | 14 and 19 | 177 |
| 21 | limit 20 to (english language and yr = “2005–Current”) | 168 |
